# Epigenetic inheritance based evolution of antibiotic resistance in bacteria

**DOI:** 10.1186/1471-2148-8-52

**Published:** 2008-02-18

**Authors:** Mike Adam, Bhuvana Murali, Nicole O Glenn, S Steven Potter

**Affiliations:** 1 Division of Developmental Biology, Children's Hospital Research Foundation, 3333 Burnet Ave. Cincinnati, OH 45229, USA

## Abstract

**Background:**

The evolution of antibiotic resistance in bacteria is a topic of major medical importance. Evolution is the result of natural selection acting on variant phenotypes. Both the rigid base sequence of DNA and the more plastic expression patterns of the genes present define phenotype.

**Results:**

We investigated the evolution of resistant *E. coli *when exposed to low concentrations of antibiotic. We show that within an isogenic population there are heritable variations in gene expression patterns, providing phenotypic diversity for antibiotic selection to act on. We studied resistance to three different antibiotics, ampicillin, tetracycline and nalidixic acid, which act by inhibiting cell wall synthesis, protein synthesis and DNA synthesis, respectively. In each case survival rates were too high to be accounted for by spontaneous DNA mutation. In addition, resistance levels could be ramped higher by successive exposures to increasing antibiotic concentrations. Furthermore, reversion rates to antibiotic sensitivity were extremely high, generally over 50%, consistent with an epigenetic inheritance mode of resistance. The gene expression patterns of the antibiotic resistant *E. coli *were characterized with microarrays. Candidate genes, whose altered expression might confer survival, were tested by driving constitutive overexpression and determining antibiotic resistance. Three categories of resistance genes were identified. The endogenous β-lactamase gene represented a cryptic gene, normally inactive, but when by chance expressed capable of providing potent ampicillin resistance. The glutamate decarboxylase gene, in contrast, is normally expressed, but when overexpressed has the incidental capacity to give an increase in ampicillin resistance. And the DAM methylase gene is capable of regulating the expression of other genes, including multidrug efflux pumps.

**Conclusion:**

In this report we describe the evolution of antibiotic resistance in bacteria mediated by the epigenetic inheritance of variant gene expression patterns. This provides proof in principle that epigenetic inheritance, as well as DNA mutation, can drive evolution.

## Background

Evolution requires phenotypic variation, selection, and heritability. It is generally assumed that mutation provides the single source of biological diversity that fuels evolution. In this report we describe evolution without mutation.

We investigated the evolution of antibiotic resistance by bacteria, a topic of some importance. Bacterial and viral infections are responsible for approximately 5–10% of deaths in industrialized nations, over 30% of deaths in Southeast Asia, and over 60% of deaths in Africa, according to the Global Health Council, and antibiotic resistant strains are becoming increasingly prevalent.

This study began as an effort to better understand the origin of satellite colonies, the small colonies that typically surround a colony of *E. coli *carrying, for example, a plasmid conferring ampicillin (amp) resistance during a subcloning experiment. It is generally stated that the colony of cells carrying the plasmid is able to reduce the concentration of amp in its vicinity, thereby allowing other cells to survive. It is interesting to note, however, that normally there will be a large number of cells immediately surrounding the resistant colony, and only a few of these cells form satellite colonies. What distinguishes these few cells and allows them to grow rather than die? In this report we show that the mechanism is not based on DNA mutation or plasmid uptake. Instead, within an isogenic population, without significant genotypic variation, there exists selectable phenotypic variation based on stochastic differences in gene expression patterns. That is, heritable epigenetic variation creates phenotypic diversity for natural selection to act upon.

## Results

### Resistance rates

To re-create satellite colony forming conditions we placed isogenic XL1-Blue *E. coli*, from a single colony, on agar plates with several different low concentrations of amp. Under our laboratory conditions the MIC (minimal inhibitory concentration, giving no detectable growth in liquid culture following an overnight incubation) was 2.5 μg/ml amp. We observed, however, that there was significant cell death even at sub-MIC antibiotic concentrations. For example, only 21% (+/-3%) of cells survived and formed colonies on plates with 1 μg/ml amp.

This simple experiment demonstrates the existence of selectable phenotypic variation in an isogenic population. A significant fraction of cells survived, while most died, similar to what is seen in the formation of satellite colonies. The key question addressed in this study is why, within a population of *E. coli*, will some individuals survive to form colonies while genetically identical neighbors die?

It is important to note that percentage of cells surviving 1 μg/ml amp, at about 20%, is too high to be accounted for by the occurrence of random spontaneous mutations. Frequencies of stable mutation to even low levels of antibiotics are far lower than this. For example, the rate of selection of stable mutant low-level quinolone resistant mutants in *P. aeruginosa *ranges from 1.2 × 10^-6 ^to 4 × 10^-10^, depending on the concentration of quinolone used [1]. While it is true that the frequency of mutation to antibiotic resistance is dependent on many variables, including the number of target genes, and the numbers of positions within those genes that can be mutated to confer resistance, nevertheless the observed high rate of 20% argues very strongly against an explanation based on stable DNA mutations.

**Figure 1:**
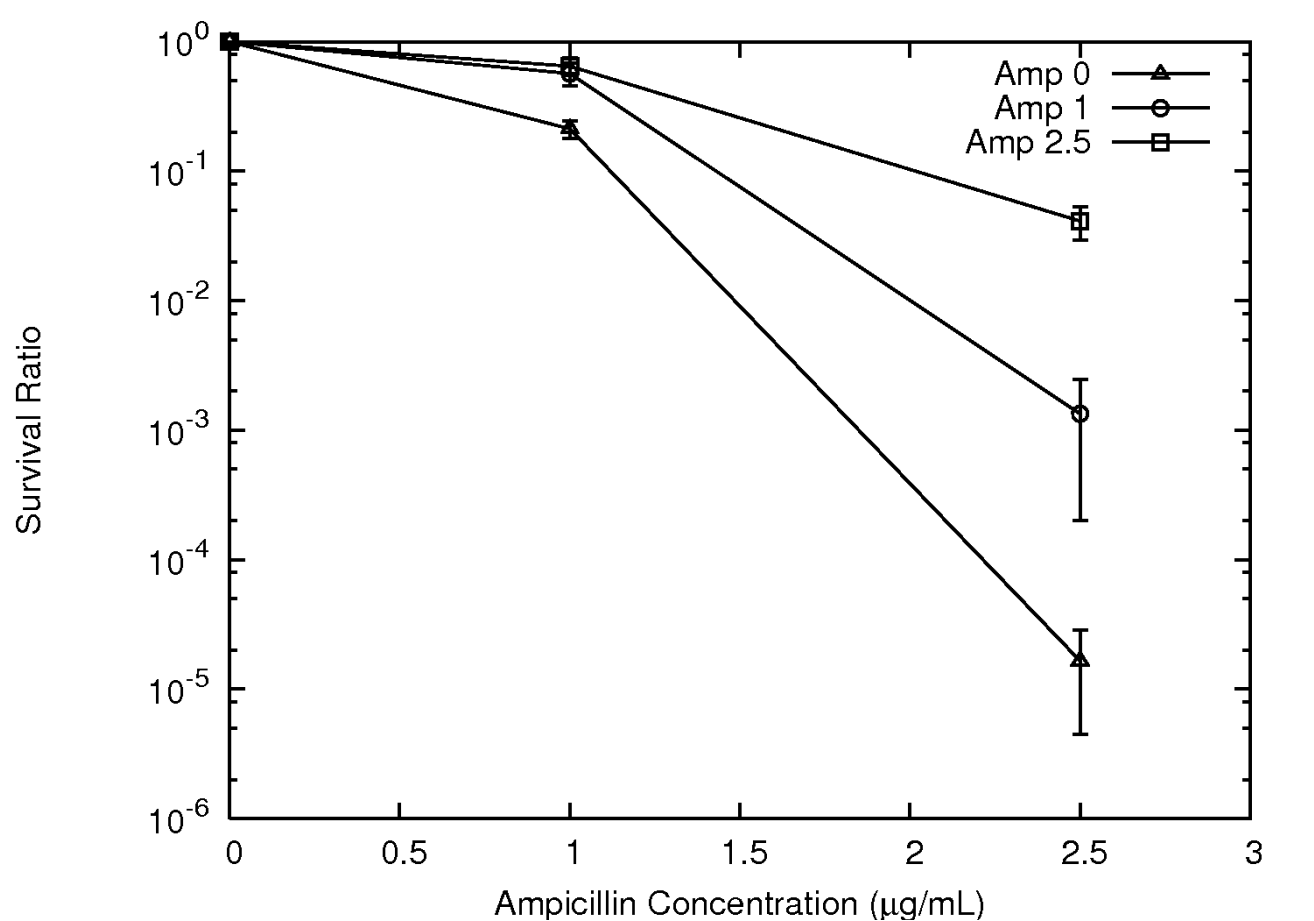
***E. coli *ampicillin survival curves**. All *E. coli *are isogenic, recently derived from a single colony. Triangles show survival rates for cells previously grown in the absence of ampicillin. Resistance rates were ramped higher by prior exposure to ampicillin. Cells surviving selection of 1 μg/ml ampicillin (circles) showed improved survival on both 1 μg/ml and 2.5 μg/ml ampicillin. Cells surviving 2.5 μg/ml ampicillin (squares), showed even more dramatic improvement, with an approximately three log increase in survival on 2.5 μg/ml ampicillin. Nevertheless, reversion rates were extremely high, with for example over 95% of cells from 2.5 μg/ml ampicillin not surviving dispersion and immediate re-plating on another LB agar plate with the same antibiotic concentration, 2.5 μg/ml ampicillin. Survival rates for previously unexposed *E. coli *at ampicillin concentrations of 5 μg/ml and higher were extremely low, below 1/10^6^.

As expected, the percent survivors dropped dramatically as the amp levels increased, with only approximately 1/10^5 ^cells surviving on the MIC concentration of 2.5 μg/ml amp (Fig. 1). Survival rates were routinely determined by performing parallel serial dilution platings on LB agar with and without antibiotic.

We next asked if the survival rate could be ramped higher by successive exposures to increasing concentrations of amp. We found that this was indeed the case. For example, cells surviving an initial exposure to 1 μg/ml amp were then found to show over 50% survival on a second plating on 1 μg/ml amp, compared to the original 20%, and cells surviving 2.5 μg/ml amp gave survival rates of ~3% when re-plated on 2.5 μg/ml, an improvement of over 1000 fold compared to the 1/10^5 ^for cells not previously exposed to amp (Fig. 1). Through such successive exposures to increasing antibiotic concentrations it was possible to evolve *E. coli *that survived on amp concentrations as high as 10 μg/ml, yet showed very high reversion rates, indicating the absence of stable mutations conferring resistance.

### Reversion rates

The high frequency of survival on low antibiotic concentrations suggested an epigenetics based resistance. Perhaps some cells within the population were by chance over expressing one or more genes that conferred low level amp resistance. Epigenetic inheritance of this amp resistance gene expression pattern would allow such a cell to divide and form a colony, while neighboring cells without this advantage would die.

To test this model we examined reversion rates. Amp resistance due to changes in DNA sequence would be extremely stable. Reversion to antibiotic sensitivity would generally require back mutation of the precise base originally altered, a very infrequent event, estimated to be on the order of 10^-9 ^or less [2]. Epigenetic inheritance, on the other hand, is based on more unstable processes, such as maintenance of certain chromatin configurations and/or DNA methylation states, and would give much higher reversion rates.

We observed that the high frequency low level amp resistance was indeed extremely unstable, showing very high reversion rates to antibiotic sensitivity.

For example, over 95% of cells taken from a single colony growing on a 2.5 μg/ml amp plate were unable to grow when immediately transferred to a new plate with the same 2.5 μg/ml amp concentration (Fig. 1). This immediate reversion rate of over 95% to amp sensitivity argues strongly in favor of an epigenetic inheritance based mechanism, and against the involvement of DNA mutation.

These results are quite striking. When the cells of a colony on a plate with 2.5 μg/ml amp were quickly resuspended in LB broth and immediately re-plated in parallel on plates with amp (2.5 μg/ml amp again), and without amp to determine viable cell count, it was observed that over 95% of the cells placed on the amp plate failed to form a colony. That is, over 95% of the cells reverted to antibiotic sensitivity over the brief time span of the experiment. It is clear from these results that this antibiotic resistant state is extremely unstable.

It is also interesting to note that this instability disappeared as the time under selection, and antibiotic concentrations were increased. The cells surviving 30 μg/ml amp showed variable but generally low reversion rates, suggesting that most of these cells had now acquired stable DNA mutations conferring amp resistance.

### Selection Scheme

A "whole plate" selection scheme was primarily used (see Methods). At each step of antibiotic selection we pooled a few hundred colonies from a single plate, by adding five ml of LB broth to the surface of the plate, scraping the cells into the LB broth, and resuspending the cells by pipeting. The cells were then immediately re-plated, in serial dilutions, in the absence of antibiotic to determine viable cell count, and on plates with different antibiotic concentrations, to create survival curves, determine reversion rates, and to select for increased antibiotic resistance.

### Variable Gene Expression

To further investigate the basis of the antibiotic resistance we examined gene expression patterns in multiple *E. coli *populations surviving selection with 0, 1, 2.5, 5, 10 or 30 μg/ml amp, using a total of 35 Affymetrix *E. coli *version 2.0 microarrays (see Methods). Altered gene expression patterns in resistant cells might reveal epigenetic based survival mechanisms.

**Figure 2:**
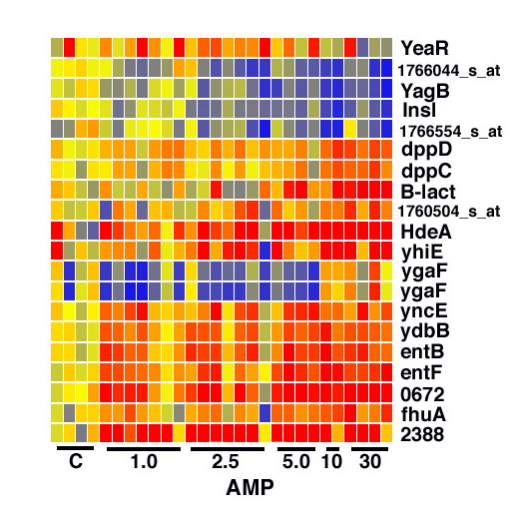
**Heat map showing changing gene expression patterns in ampicillin resistant *E. coli***. Each column represents one population grown in the absence of amp (C), or in the concentration of amp shown at the bottom. Each row shows the expression level across populations for one gene, with blue for low, red for high and yellow for intermediate expression. The endogenous *β-lactamase *gene, AmpC, shows elevated expression in most, but not all, ampicillin resistant populations.

Biological replicates were used for the microarray analysis, with each microarray representing an independently generated biological sample. The data was first examined with GeneSpring software, performing an ANOVA analysis, producing a list of differentially expressed genes. A heat map provides a visual display of the results (Fig. 2). There is some scatter in the data, perhaps reflecting the inherent variability in the selection response and the possible existence of multiple resistance mechanisms.

**Figure 3:**
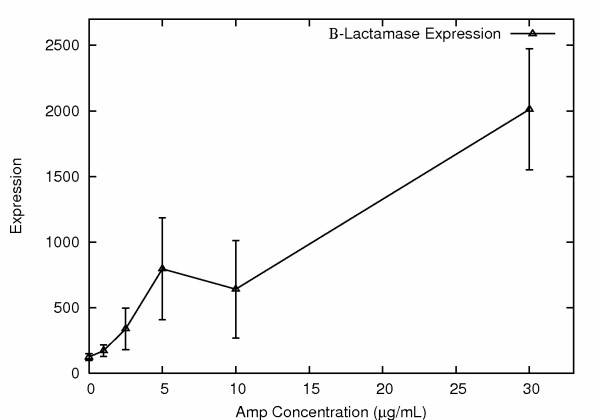
**Expression levels of endogenous *β-lactamase *gene in *E. coli *surviving selection at different ampicillin concentrations**. Transcript abundances were determined using a total of 29 Affymetrix *E. coli *2.0 oligonucleotide microarrays. Expression levels are in arbitrary Affymetrix expression units. Survivors at higher ampicillin concentrations show significantly increased *β-lactamase *expression.

Of particular interest, the list of genes with differential expression included an endogenous *E. coli *gene with known β-lactamase activity. This gene, AmpC, is present in most laboratory strains of *E. coli*. A close relative of *E. coli*, *E. cloacae*, carries an AmpR gene important in the induction and repression of AmpC. It has been shown that the AmpR gene was deleted from the *E. coli *genome following the divergence of *E. coli *and *E. cloacae *from their common ancestor [3]. This results in very low-level constitutive expression of AmpC in *E. coli*, allowing the use of amp selection in cloning experiments, for example. Amp resistance in *E. coli *has previously been associated with stable DNA mutations resulting in novel AmpC promoters, weakened attenuators or AmpC gene duplications [4-8]. Of interest, however, we observed elevated expression of AmpC not only in the stable amp resistant populations (resistant to 30 μg/ml amp), but also in many of the unstable amp resistant populations (resistant to 1, 2.5, 5 and 10 μg/ml amp) (Fig. 3).

These results suggest that AmpC can contribute to amp resistance through three distinct mechanisms. In *E. cloacae *the presence of amp can induce elevated AmpC expression through the action of the AmpR gene. That is, the combination of the AmpR and AmpC genes provide a survival mechanism that is induced in the presence of amp. In *E. coli*, without the AmpR gene, DNA mutations can result in elevated AmpC expression and stable amp resistance, as shown previously. In addition, in this report, we show that unstable resistance to low levels of amp in *E. coli *can be mediated through epigenetic events, likely stochastic in nature, yet semi-stable, that result in elevated AmpC expression.

To test for functionality in conferring amp resistance we picked a total of eight genes with elevated expression following amp selection, after examining the microarray data, and subcloned their coding sequences into an expression vector plasmid, which was then introduced into *E. coli*. The over-expression of the endogenous *E. coli *AmpC gene did indeed confer dramatically increased amp resistance, as expected, allowing all cells to survive on up to 30 μg/ml amp, but not 100 μg/ml. None of the other genes tested (see Methods) gave significant survival benefits. These results argue that the observed increase in expression of the endogenous AmpC gene was responsible, at least in some cases, for the observed amp resistance.

It is not surprising that many of the genes with elevated expression following amp selection were unable to confer amp resistance. It has been previously shown that very low, subinhibitory concentrations of antibiotics can cause significant changes in bacterial gene expression patterns. For example, it was shown in *Salmonella typhimurium*, using a promoter-Lux reporter library, that many promoters responded to low levels of erythromycin and rifampicin, below the MIC [9].

In an attempt to remove some of the induced genes unrelated to antibiotic resistance from the list of differentially expressed genes we re-analyzed the microarray data, only this time including the data from *E. coli *exposed to very low levels of amp (1 μg/ml) in the control group. This more specifically looked for genes that showed increasing change in expression concordant with survival in the presence of increasing levels of amp. A pairwise t-test was performed using GeneSifter software. The top five genes on the resulting list, ranked by fold change, were as follows. (1.) Glutamate decarboxylase (GadA), up 7.2 fold, p = 0.021. (2.) The second gene on the list encodes a separate glutamate decarboxylase (GadB) up 6.1 fold, p = 0.014. It is interesting that the top two genes on the list are located at very different genomic positions, yet encode isozymes very similar in sequence and function. (3.) The third gene, GadC, encodes an APC transporter that mediates export of gamma-aminobutyrate in exchange for glutamic acid. The GadC gene is located on the same operon as GadB, providing a measure of cross-validation. (4.) The fourth gene, AmpC, was up-regulated 4.4 fold, with a p value of 0.0034 (the lowest p value). (5.) The fifth gene, hdeB, was up 4.0 fold, p = 0.011, and encodes an acid stress chaperone.

**Figure 4:**
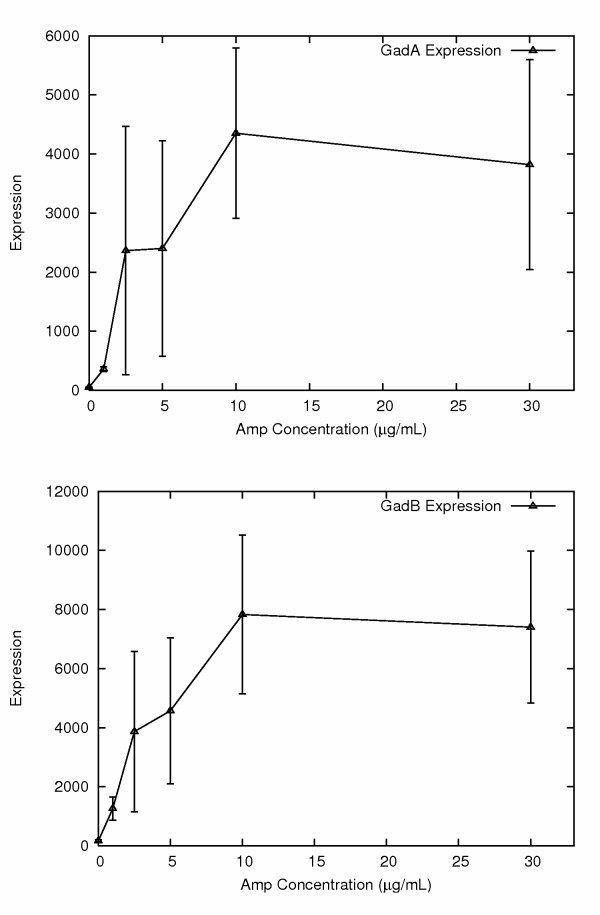
**Elevated expression of *GadA *and *GadB *genes in ampicillin resistant populations**. Both genes encoding the two glutamate decarboxylase isozymes show significantly elevated expression in ampicillin resistant *E. coli *populations.

**Figure 5:**
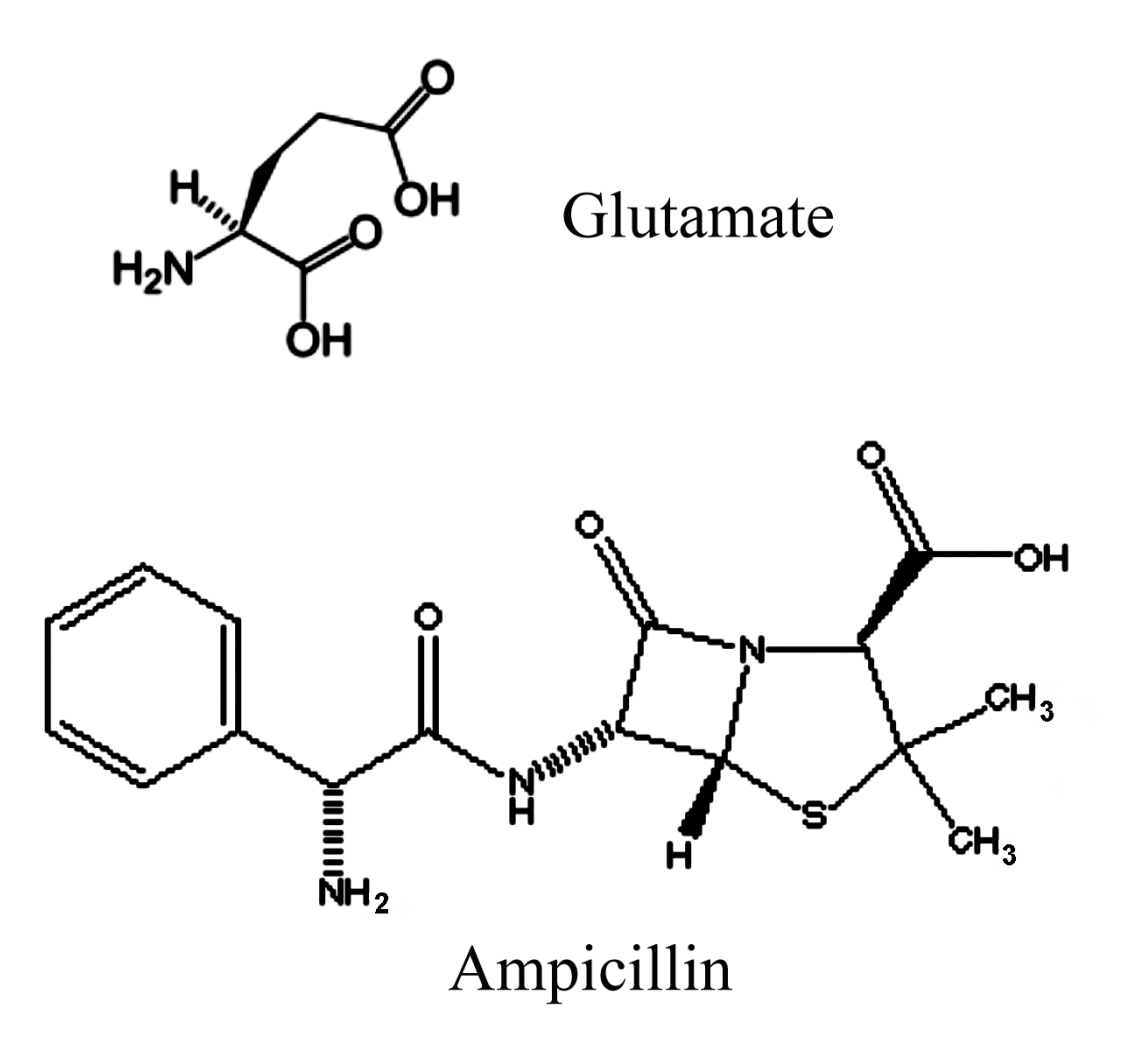
**Structures of glutamate and ampicillin**. Similarities between the lactam ring and the peptide backbone have been proposed to be responsible for the antibiotic function of ampicillin.

It is quite interesting that the two genes showing the greatest upregulation in more amp resistant cells encode isozymes that both decarboxylate glutamate (Fig. 4). There are reasons to suspect that this overexpression of glutamate decarboxylase could be functionally related to amp resistance. Glutamate decarboxylase removes a carboxyl group from glutamic acid (which is neuro-excitatory in mammals), producing gamma-aminobutyric acid (GABA, neuro-inhibitory in mammals). There is a surprising connection between penicillin and glutamate-GABA in mammals. Penicillin is a GABA_A _receptor blocker and can be used to induce convulsions in animal models of epilepsy [10-12]. This ability of penicillin to interact with a GABA receptor suggests a steric similarity between penicillin and glutamate-GABA. Of interest, both penicillin, (as well as ampicilliin), and glutamate do carry a carboxyl group in a comparable chemical setting (Fig. 5). Indeed the structural similarity between a region of ampicillin (including the carboxyl group) and the backbone of a peptide chain have been used to explain the mechanism of action of β-lactam antibiotics. This suggests that glutamate decarboxylase might be capable of removing the carboxyl group from ampicillin, thereby inactivating it.

Previous work also suggests another possible role for glutamate decarboxylase in antibiotic resistance. The decarboxylation reaction consumes a proton, contributing to membrane potential difference, which in turn is used by the AcrAB multi-drug efflux pump [13]. The overexpression of the GadA and GadB genes may therefore provide increased power to the AcrAB pump.

To test GadA for possible function in providing amp resistance we made a GadA expression plasmid and introduced it into the XL1-Blue *E. coli*. We observed that the XL1-Blue *E. coli *with the GadA expression construct showed somewhat improved survival rates, five fold higher colony formation on 1.75 μg/ml amp compared to cells without (P = 0.014). These results suggest that over expression of GadA, and likely the isozyme GadB, can contribute to survival under amp selection conditions. Therefore the increased expression of GadA and GadB could be the result of stochastic variation in expression levels of these genes coupled with amp selection for cells with higher expression. It is also interesting to note the possibility that just as penicillin can perturb glutamate signaling in the mammalian brain, perhaps through its structural similarity to glutamate, penicillin (or ampicillin) can induce GadA and GadB expression in *E. coli*.

### Nalidixic Acid and Tetracycline

To study the epigenetic based evolution of antibiotic resistance further, and in particular to determine if this was a general phenomenon or restricted to ampicillin, we performed similar selection experiments using two additional antibiotics, nalidixic acid and tetracycline. These three antibiotics exhibit distinct modes of action, with ampicillin disrupting cell wall biosynthesis, nalidixic acid inhibiting DNA synthesis and tetracycline blocking protein synthesis.

**Figure 6:**
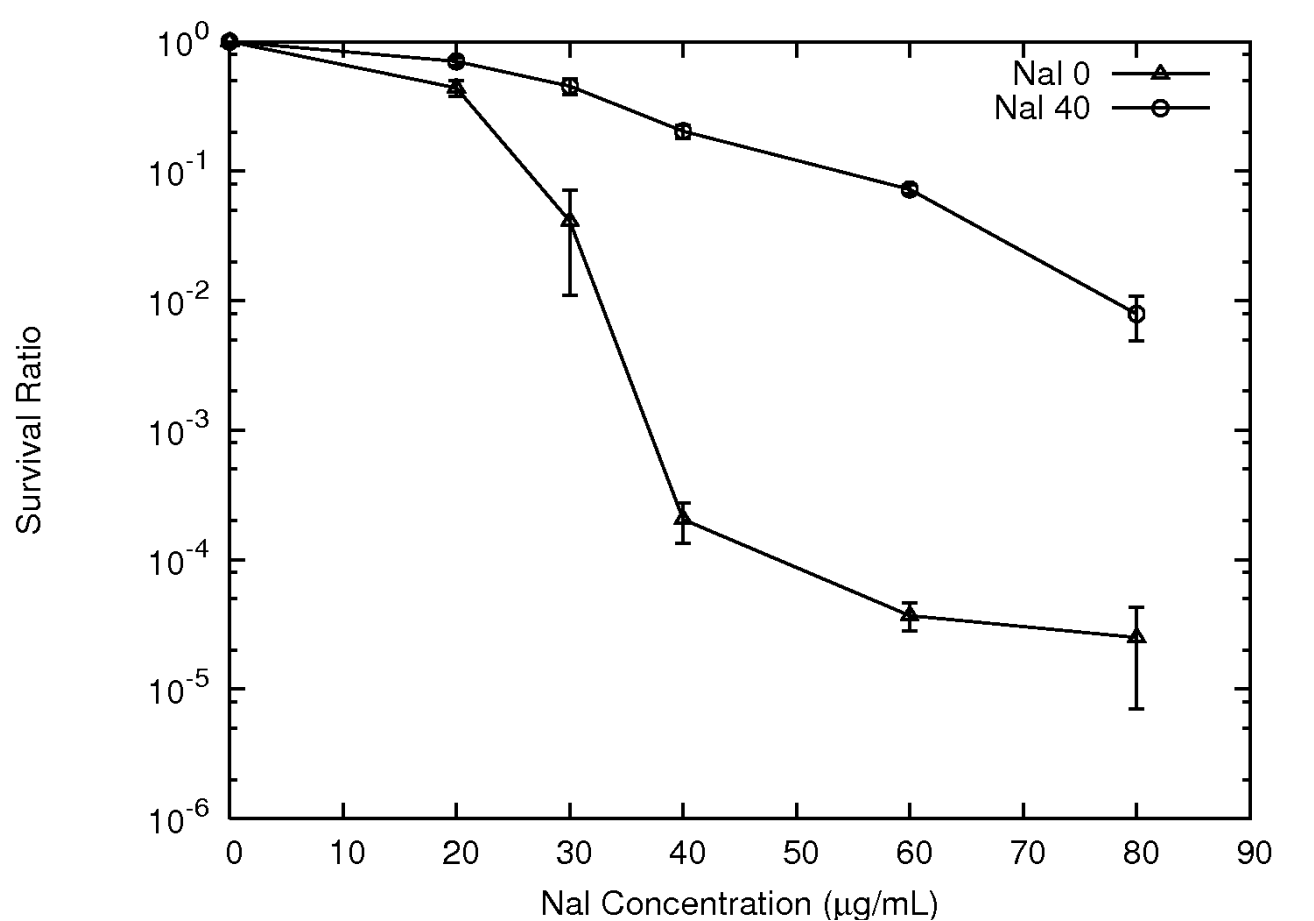
***E. coli *nalidixic acid survival curves**. As observed for ampicillin, the survival rates at low nalidixic acid concentrations are too high to be accounted for by spontaneous mutation. Cells surviving prior antibiotic exposure show dramatically improved resistance rates, yet reversion to antibiotic sensitivity is very common, with over 95% of cells from 40 μg/ml nalidixic acid not surviving re-exposure to the same antibiotic concentration. No previous nalidixic acid exposure, triangles. From 40 μg/ml nalidixic acid, circles.

Interestingly, the results for the nalidixic acid and tetracycline selection experiments closely mirrored those observed using ampicillin, showing the generality of epigenetic inheritance mediated evolution of antibiotic resistance. For example, again starting with isogenic XL1-Blue *E. coli*, we established a nalidixic acid survival curve, finding survival rates that varied from about 40% on 20 μg/ml nalidixic acid to about 1 in 100,000 on 80 μg/ml nalidixic acid (Fig. 6). For reference, the nalidixic acid MIC for XL1-Blue *E. coli *was 40 μg/ml. As for ampicillin, the survival rates under low concentration antibiotic concentration selection were too high to be accounted for by spontaneous mutation. This was confirmed by testing reversion rates, which with rare exception were again very high, indicating that the resistance was not the result of a stable change in DNA sequence (Fig. 6).

The XL1-Blue *E. coli *carry a stable mutation in the gyrase gene (gyrA96), conferring significantly greater nal resistance than found in wild type *E. coli*, which typically would not survive even 10 μg/ml nalidixic acid. In this series of experiments, therefore, we started with *E. coli *showing moderate levels of nal resistance, and examined the evolution of increased resistance to still higher antibiotic concentrations.

Once again the resistance rates could be elevated by successive antibiotic selection. For example, upon initial exposure to 40 μg/ml nalidixic acid approximately 1/1,000 cells survived to form a colony. When cells from a 40 μg/ml nalidixic acid plate, however, were re-plated on another plate with 40 μg/ml nalidixic acid, then about 20% survived to form colonies, showing a dramatic improvement in survival rate. This also illustrates the high reversion rate, as 80% of cells do not survive a re-plating on 40 μg/ml nalidixic acid.

The ability to ramp antibiotic resistance higher by successive exposures was also illustrated by the survival rates on higher concentrations of nalidixic acid. For example, upon initial exposure to 80 μg/ml nalidixic acid only about one cell in 100,000 survived. But if cells surviving exposure to 40 μg/ml were subsequently selected at 80 μg/ml then almost 1 cell in 100 survived, approaching a 1,000 fold improvement (Fig. 6).

**Figure 7:**
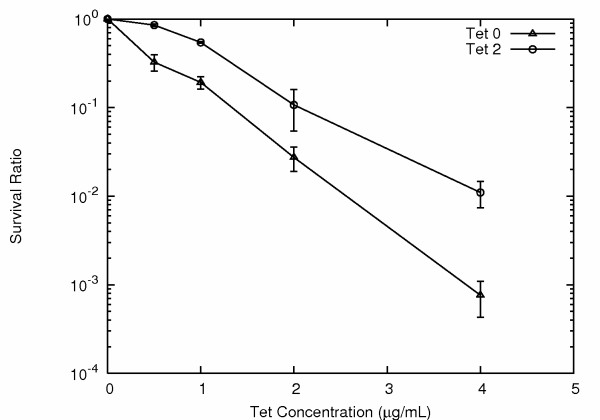
***E. coli *tetracycline survival curves**. Survival ratios for *E. coli *not previously exposed to tetracycline (triangles), and for *E. coli *previously surviving exposure to 2 μg/ml tetracycline (circles).

For the tetracycline selection experiments we used a different strain of *E. coli *[XL1-Blue (MRF')], as the standard XL1-Blue cells carry genes conferring strong tet resistance. The XL1-Blue (MRF') cells (Stratagene) are closely related to XL1-Blue cells (see Methods), only with kanamycin resistance instead of tetracycline resistance. The results of tetracycline selection using single colony derived isogenic XL1-Blue (MRF') cells showed a similar pattern to that observed for both ampicillin and nalidixic acid. The tetracycline MIC for XL1-Blue (MRF') *E. coli *was 1.0 μg/ml. Once again, resistance and reversion rates were far too high to be accounted for by stable DNA mutations, and once again it was possible to ramp up resistance rates by successive exposures to increasing concentrations of antibiotic (Fig. 7).

### Molecular Mechanisms

The observed semi-stable epigenetic inheritance could be mediated by DNA methylation [14], chromatin modifications, superhelical domain configuration [15], or perhaps other mechanisms. DNA methylation in particular provides a plausible mechanism for the observed metastable antibiotic resistance. The *E. coli *deoxyadenosine methyltransferase (DAM), for example, methylates the adenine of the GATC sequence. This sequence occurs approximately 18,000 times in the *E. coli *genome, and the two copies of this palindromic sequence opposite each other on DNA are generally both methylated. Following DNA replication, however, the DNA is transiently hemimethylated, and the new strand is then methylated by DAM.

For a few sites in the *E. coli *genome, however, the methylation status of the DAM target, GATC, is variable and can impact gene expression. Of particular interest, the *flu *gene is a metastable locus encoding the Ag43 protein, which is an outer membrane protein promoting cell-cell aggregation important in biofilm production. Phase variation, in both directions (Ag43+ to Ag43- and vice versa), is metastable and occurs with a frequency of approximately 10^-3 ^[16]. DAM and OxyR repressor compete for binding to sequences in the *flu *promoter, with OxyR binding resulting in repression and blocking of methylation, while DAM methylation is required for full gene activation [17].

A previous microarray analysis of gene expression patterns associated with biofilms showed a strong upregulation of the *flu *gene [18]. Indeed, it was shown that *E. coli *with a mutation of this gene were unable to form biofilms [18]. Because of the metastable binary expression of this gene, and its strong association with biofilms, and the known multidrug antibiotic resistance of biofilm *E. coli *compared to *E. coli *undergoing planktonic growth, we were particularly interested in the expression levels of the *flu *gene in the antibiotic resistant *E. coli *in this study. Interestingly, we observed no change in *flu *gene expression in the amp resistant cells, arguing against the involvement of a biofilm type genetic program in the antibiotic resistance.

Although the elevated expression of the *flu *gene, and consequent biofilm production, did not appear associated with the antibiotic resistance described in this report, nevertheless the DNA methylation mediated metastable expression of the *flu *gene provided a useful model for possible regulation of other genes that might indeed be responsible for the observed selectable antibiotic resistance.

Only a small number of genes were shown to be altered in expression in Dam methylation mutant *E. coli *[19]. The 18 genes with over 2 fold change in DAM mutant cells included representatives of the Csg and Mar operons, which were both down regulated in the absence of DAM methylation.

To test the possible function of DNA methylation in the observed evolution of antibiotic resistance we examined the ER2925 strain of *E. coli *K12, which is deficient for both DAM and DCM dependent DNA methylation. We generated both DAM positive and DCM positive variants of the ER2925 cells by making DAM and DCM expression plasmids, allowing a direct comparison of cells with and without these specific DNA methylases. We observed that DCM had no apparent effect on antibiotic survival, while DAM methylase did improve the survival rate by a factor of five on nalidixic acid (40 μg/ml).

## Discussion

**Figure 8:**
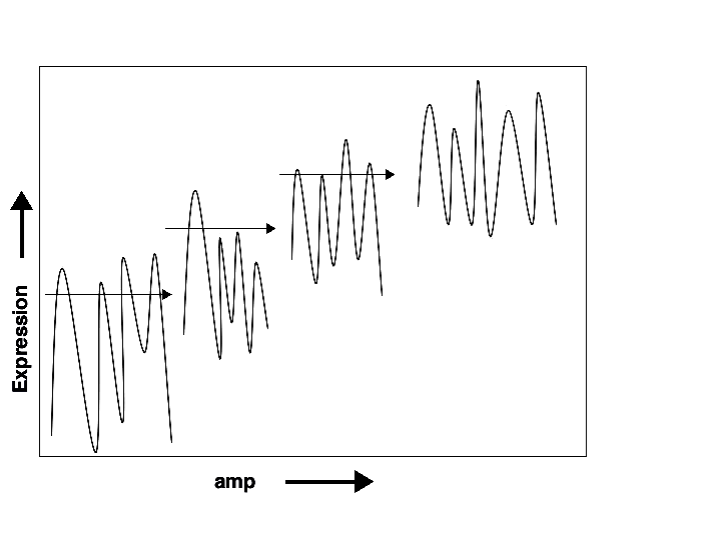
**Model of evolution of antibiotic resistance through epigenetic inheritance**. Within an isogenic population of *E. coli *there is random variation in expression levels of genes. Antibiotic exposure (horizontal arrows) selects cells with gene expression patterns that allow survival. For example, elevated expression of the *GadA *and *β-lactamase *genes promote ampicillin survival. A combination of continued selection, epigenetic inheritance and stochastic variation can evolve populations with gene expression patterns providing increasing antibiotic resistance.

In this report we present data strongly supporting the conclusion that evolution can occur without mutation. A model to explain the observed evolution of antibiotic resistance is illustrated in Fig. 8. Within an isogenic population of *E. coli *there is random variation in the expression levels of genes, creating phenotypic variation. This epigenetic variation shows an element of heritability. Some cells with elevated expression of genes conferring antibiotic resistance survive antibiotic selection, as do sufficient progeny to allow colony formation.

The cells with increased expression of resistance genes in turn show variation in expression levels, but now under selection and therefore with sustained elevated levels, allowing selection of still higher degrees of resistance. Serial exposure to rising concentrations of antibiotic results in cells with higher resistance than possible with a single round of selection (Fig. 8).

We show that there are several categories of resistance genes. The endogenous *E. coli *AmpC gene represents a cryptic gene, normally not significantly expressed, but when activated capable of conferring potent antibiotic resistance. The glutamate decarboxylase genes normally provide an acid resistance function, allowing *E. coli *to transit the acidic conditions of the stomach. Elevated glutamate decarboxylase gene expression can also somewhat improve survival in the presence of ampicillin, likely incidental to its normal function. In addition there are genes like DAM that can improve survival by regulating the expression of other genes.

The evolution of resistance within the population is driven by a powerful antibiotic selective pressure coupled with phenotypic diversity created by heritable epigenetic variation. The extreme instability of the observed low level antibiotic resistance argues strongly that it does not involve DNA mutation events, or induced gene expression survival mechanisms.

### Bacteria, antibiotics and microarrays

It is important to note that several interesting previous studies examined the global effects of antibiotics on bacteria, often using microarrays [20-31]. Much of this work investigated mechanisms of antibiotic action and resistance, with the goal of discovering novel antimicrobial drugs. In some cases the survival benefits of antibiotic induced gene expression patterns are discussed [31,32]. A simple example would be the presence of tetracycline inducing the expression of a tetracycline resistance operon. Similarly, antibiotics causing DNA damage can induce an SOS DNA repair response [22,23,25].

These studies do not, however, address the issue of how epigenetic inheritance of stochastic variations in gene expression levels can provide survival advantage under antibiotic selection, as examined in this report. An example is our observation that epigenetic inheritance of elevated expression of AmpC, a gene not normally expressed in *E. coli*, significantly improves survival in the presence of ampicillin.

### Persisters

The phenomena described in this report are also distinct from the previously described bacterial persisters. For example, when *E. coli *K12 are exposed to a high concentration of ampicillin, 100 μg/ml, a small fraction of the cells, 10^-5 ^to 10^-6^, can survive, and are referred to as persisters [33]. These persisters, however, do not grow in the presence of the antibiotic, but only after its removal, and the progeny of persisters do not show an increased resistance to antibiotic. All three aspects of persisters, very low frequency, failure to grow in the presence of the antibiotic, and no increased resistance of progeny, distinguish persisters from the low level antibiotic resistant bacteria described in this report.

### Transcriptional state memory

We suggest that the observations described in this report are the result of two competing processes. On the one hand, there is noise, or stochastic variation in gene expression patterns, even within an isogenic population. This has been well documented in both bacteria and eukaryotes [34-37] and is thought to be the result in part of the probabilistic nature of mass action, with for example small numbers of transcription factors and promoter elements interacting in a single cell. On the other hand, once a gene expression pattern is established there are epigenetic memory mechanisms capable of preserving the gene expression state for multiple generations. Transcriptional memory can be mediated by DNA methylation patterns, as discussed previously, or by inherited chromatin modifications, or it can be a property of the genetic regulatory network. It is interesting to note, for example, that early studies of the lac operon showed that expression was generally quantal in nature, being on or off, depending on inducer concentration, but that at intermediate concentrations the expression state depended on the history of the cell and was stable for many generations [38].

Our results are consistent with the presence of cooperative effects in the amp resistant colonies. It is likely that some cells within the colony degrade amp through elevated AmpC expression, thereby promoting survival of all cells within the colony, and explaining why many cells within the colony appear to be antibiotic sensitive when assayed singly on a fresh antibiotic plate.

### Medical significance

The epigenetic inheritance based evolution described in this report could be of medical importance, by allowing a subpopulation of bacteria within a person to survive low doses of antibiotic. The low dose exposure could be from inappropriate antibiotic administration, from a food source, or through a protected microenvironment within the body. The cells with low-level resistance could then produce sufficient numbers for a sufficient period of time to accumulate more stable DNA mutations that confer a higher level antibiotic resistance.

## Conclusion

In summary we describe an example of an isogenic population showing selectable phenotypic variation, mediated by epigenetic inheritance. It is interesting to note that many examples of epigenetic inheritance between generations have been reported in metazoans as well, including paramutation in plants [39], the FAB-7 DNA element in Drosophila [40], and RNA-mediated epigenetic inheritance in the mouse [41]. It is reasonable to suppose that such mechanisms generating heritable phenotypic variation could provide the substrate for the action of natural selection.

## Methods

### *E. coli *strains

For amp and Nal experiments strain XL1-Blue *E. coli *(Stratagene) (recA1 endA1 gyrA96 thi-1 hsdR17 supE44 relA1 lac [F' proAB lacI^q^ZΔM15 Tn10(Tet^r^) were streaked on an LB agar plate and a single resulting colony was used for inoculation of a 50 ml LB liquid culture, which was grown to mid-log phase and then used to make one set of frozen aliquots, which were used for all of the amp and nal resistance studies. The frozen aliquots were thawed, grown in LB culture to mid-log phase, and subjected to amp or nal antibiotic selection on agar plates. For the tetracycline resistance experiments we use XL1-Blue (MRF') cells (Stratagene) with the following genotype Δ(mcrA)183Δ(mcrCB-hsdSMR-mrr)173 endA1 supE44 thi-1 recA1 gyrA96 relA1 lac[F' proAB lacI^q ^ZΔM15 Tn5(Kan^r^]. For testing the role of DNA methylation we used dam-, dcm-ER2925 *E. coli *from New England Biolabs (ara-14 leuB6 fhuA31 lacY1 tsx78 glnV44 galK2 galT22 mcrA dcm-6 hisG4 rfbD1 R(zgb210::Tn10)TetS endA1 rpsL136 dam13::Tn9 xylA-5 mtl-1 thi-1 mcrB1 hsdR2).

### MIC determination

A 2 ml LB culture was inoculated from frozen stocks for each of the cell types and grown to log phase. Approximately 10^5 ^cells were inoculated into each tube of a two fold serial dilution of each antibiotic in LB. The cultures were grown two days at 37°C in a shaking incubator. The MIC was determined to be the lowest concentration of antibiotic resulting in no turbidity, or apparent growth of the culture.

### Selection protocols

We primarily used a "whole plate" selection scheme. Log phase XL1-Blue cells were plated on LB only, and on LB plates with varying antibiotic concentrations. The cells from a few hundred colonies on a low antibiotic concentration plate were then pooled, and again plated on LB only, for titering cell concentration, as well as on plates with varying antibiotic concentrations, to determine survival rates, reversion rates, and to increase antibiotic resistance. This process was repeated with increasing antibiotic concentrations. For example, cells not previously exposed to antibiotic were first grown on 1 μg/ml amp agar plates, a few hundred colonies of surviving cells were pooled, resuspended in LB broth and plated on agar without antibiotic to determine viable cell concentration, on another plate with 1 μg/ml amp to determine reversion rate, and on plates with higher concentrations of amp to determine the survival curve and to select for higher amp resistance levels.

We found that this selection procedure eventually exposed sufficient numbers of *E. coli *for enough time to allow the accumulation of stable antibiotic resistance mutations, typically at 30 μg/ml amp.

Gene expression profiles of cells surviving 0 (three microarrays), 1.0 μg/ml amp (four microarrays), 2.5 μg/ml amp (five microarrays), 5.0 μg/ml amp (four microarrays), 10.0 μg/ml amp (two microarrays), 20.0 μg/ml amp (one microarray) and 30.0 μg/ml amp (four microarrays) were determined.

The variable sizes of the resistant colonies suggested the presence of different growth rates, and different levels of antibiotic resistance among individual colonies. To reduce the numbers of *E. coli *exposed to selection, to reduce the incidence of stable mutations, and to begin to define the individual differences among colonies, we also tested a single colony selection scheme, using single colonies from selection plates instead of pooling hundreds of colonies. Because of the smaller numbers of cells involved, however, we were unable to select populations of *E. coli *with greater than 5 μg/ml amp resistance. The populations generated were also subjected to microarray analysis, using three arrays each to examine expression profiles of cells surviving 1.0 and 2.5 μg/ml amp.

A third selection scheme used concentrated cell streaks instead of single colonies, emphasizing the community effects on survival. Expression profiles were determined for cells surviving 1.0 μg/ml amp (two microarrays), 2.5 μg/ml amp (two microarrays), 5.0 μg/ml amp (two microarrays) and 10.0 μg/ml amp (three microarrays).

### Assaying genes for antibiotic resistance function

The amp resistance gene in the pl451 plasmid was removed by restriction digestion with *Bsa*I and *Xmn*I, blunting with Klenow, and ligation. A *Sca*I restriction site was introduced immediately downstream of the promoter of the deleted amp gene using the QuickChange Site-Directed Mutagenesis Kit (Stratagene, La Jolla, CA) and the primer pair GGGCGACACGGAAATGTTGAATACTAGTACTCTTCCTTTTTC, GAAAAAGGAAGAGTACTAGTATTCAACATTTCCGTGTCGCCC.

The coding sequences of the genes to be tested were amplified by high fidelity PCR and used for in vivo subcloning into the linearized Sca1 site of pl451 using the Xi-Clone High Speed Cloning Kit (Genlantis, San Diego, CA). The resulting constructs showed no signs of instability. Genes were assayed for amp resistance function in XL1-Blue cells. Primers used for amplification were as follows:

YedH (multi drug resistance pump) ATGCTTCAATAATATTGAAAAAGGAAGAGTGTGCAGAAGTATATCAGT and AAGGGCGACACGGAAATGTTGAATACTAGTTTAGCGGGATGCTCGTTG,

bdb 945215

ATGCTTCAATAATATTGAAAAAGGAAGAGTATGATCTGGAAACGCCATTTA, AATAAGGGCGACACGGAAATGTTGAATACTAGTTCATCCCAAAACTGCCG,

YedX

TGCTTCAATAATATTGAAAAAGGAAGAGTATGTTAAAGCGTTATTTAGTA, AAGGGCGACACGGAAATGTTGAATACTAGTTTAACTGCCACGATAGGT,

Z0726

TGCTTCAATAATATTGAAAAAGGAAGAGTATGGCATTCAGTAATCCC,

AATAAGGGCGACACGGAAATGTTGAATACTAGTTCATTGTGCCTCCTGCA, 2-isopropylmalate synthase (LeuA)

TAAATGCTTCAATAATATTGAAAAAGGAAGAGTATGAGCCAGCAAGTC,

AAGGGCGACACGGAAATGTTGAATACTAGTTCACACGGTTTCCTTGTTGTTT, Sulfate adenyltransferase subunit1

TAAATGCTTCAATAATATTGAAAAAGGAAGAGTATGAACACCGCACTTGCACA,

AATAAGGGCGACACGGAAATGTTGAATACTAGTTTATTTATCCCCCAGCAAATC,

Sulfate adenyltransferase subunit2

TAAATGCTTCAATAATATTGAAAAAGGAAGAGTATGGATCAAATACGAC,

AATAAGGGCGACACGGAAATGTTGAATACTAGTTTAAAAATACCCCTGACGTT

β-lactamase (AmpC) TGCTTCAATAATATTGAAAAAGGAAGAGTATGTTCAAAACGACGCTCTG, AATAAGGGCGACACGGAAATGTTGAATACTAGTTTACTGTAGAGCGTTAAGA.

DAM

5-GCTTCAATAATATTGAAAAAGGAAGAGTATGAAGAAAAATCGCGCTTT-3

5-AAGGGCGACACGGAAATGTTGAATACTAGTTTATTTTTTCGCGGGTGAAA-3

DCM

5-TGCTTCAATAATATTGAAAAAGGAAGAGTATGCAGGAAAATATATCAGTA-3

5-AGGGCGACACGGAAATGTTGAATACTAGTTTATCGTGAACGTCGGCCATG-3

GadA

5-TGCTTCAATAATATTGAAAAAGGAAGAGTATGGACCAGAAGCTGTTAACG-3

5-AGGGCGACACGGAAATGTTGAATACTAGTTCAGGTGTGTTTAAAGCTGTT-3

### Microarray analysis

We harvested *E. coli *(XL1-Blue) from agar plates with 1000–2000 colonies by adding 5 ml of LB broth and scraping to resuspend. Approximately 10^9 ^cells were pelleted and RNA was isolated using the RNeasy mini prep kit (Qiagen, Valencia, CA). cDNA was synthesized, fragmented, labeled, and hybridized to microarrays as per the Affymetrix prokaryotic sample and array processing protocol (Affymetrix, Inc., Santa Clara, CA).

## Authors' contributions

MA determined ampicillin survival ratios, with help from NG, and subcloned genes into expression plasmids for antibiotic resistance functional analysis and helped write paper. BM performed tetracycline survival ratio determinations. SP conceived experiments, determined nalidixic acid survival ratios and helped write paper.
